# Strange themes in pandemic dreams: Insomnia was associated with more negative, anxious and death‐related dreams during the COVID‐19 pandemic

**DOI:** 10.1111/jsr.13655

**Published:** 2022-06-14

**Authors:** Hailey Meaklim, Malisa Burge, Flora Le, Sukjhit K. Bains, William Saunders, Stephen Ghosh, Moira F. Junge, Prerna Varma, Imogen C. Rehm, Melinda L. Jackson

**Affiliations:** ^1^ Turner Institute for Brain and Mental Health, School of Psychological Sciences Monash University Monash Victoria Australia; ^2^ The Sleep Health Foundation Blacktown New South Wales Australia; ^3^ College of Health and Biomedicine Victoria University Melbourne Victoria Australia

**Keywords:** acute insomnia, dreaming, longitudinal study, mental health, mixed‐methods, thematic analysis

## Abstract

Dreaming and insomnia are important markers of distress in times of crisis. Here, we present a longitudinal, mixed‐methods study examining changes in dreaming between individuals with and without insomnia symptoms and their relationship to mental health during the COVID‐19 pandemic. A global survey examining insomnia symptoms, dreams and mental health was launched in April 2020 and followed participants over 12 months. Of 2240 participants, 1009 (45%) reported dream changes at baseline. A higher proportion of participants with new‐onset insomnia reported dream changes (55%) than those with pre‐existing insomnia (45%) or good sleepers (36%). Overall, thematic analysis identified key dream change themes of increased dream activity, with participants dreaming vividly, in high‐definition, and with a strong negative charge. Themes around survival, adjusting to pandemic life, meaning‐making and poor sleep quality were also noted. Linguistic Inquiry Word Count showed that individuals with insomnia used more negative words to describe their dream changes than good sleepers. Specifically, the new‐onset insomnia group used more anxious and death‐related words than those who slept well. Notably, all groups experienced a significant reduction in dream activity by 3‐month follow‐up. Lastly, dream changes were associated with worse mental health symptoms over time, and this effect was more pronounced in individuals with insomnia. Our results highlight that insomnia symptoms, especially new‐onset insomnia, are associated with more negative dream changes during collective stressful events, potentially compounding daytime distress and mental health symptoms over time. During times of crisis, dreaming and insomnia may reveal an important target for mental health interventions.

## 1 INTRODUCTION

Dreaming is described as a simulation of waking life, allowing for reflection on conscious thoughts, emotions and experiences (Domhoff, 1996; MacKay & DeCicco, 2020; Revonsuo, Tuominen, & Valli, 2015). The continuity hypothesis of dreaming contends that dream content is continuous with changes in our waking day experiences (Domhoff, 1996). As a result, stressful or threatening events during wakefulness can lead to more frequent and severe threat‐based dreams during sleep (Revonsuo, 2000). For example, changes in dreaming and nightmare frequency have been recorded after collective stressful events throughout history, including earthquakes, wars and terrorist attacks (Barrett et al., 2013; Schredl & Piel, 2006; Tang, Lu, Yang, & Xu, 2018). Increased nightmare frequency can persist for many years, and is associated with elevated symptoms of insomnia, depression and anxiety long‐term (Barrett et al., 2013; Sandman et al., 2013; Solomonova et al., 2021). Collective stressful or threatening events, therefore, present a unique opportunity to study changes in dream experiences and their relationship with insomnia symptoms and mental health.

The COVID‐19 pandemic created increased distress and disruption to daily routines around the world. Consequently, the pandemic has been associated with increases in dream and nightmare frequency (Gorgoni et al., 2021; Pesonen et al., 2020; Scarpelli et al., 2021). Waking day concerns of COVID‐19 have affected dream imagery (MacKay & DeCicco, 2020), dream recall frequency (Schredl & Bulkeley, 2020), dream content (Iorio, Sommantico, & Parrello, 2020; Mota et al., 2020; Pesonen et al., 2020) and emotional tone of dreams (Iorio et al., 2020; Mota et al., 2020; Schredl & Bulkeley, 2020). Pandemic dreams are associated with increased death‐related content, lower positive emotional tone and higher negative emotional tone when compared with pre‐pandemic dreams (Barrett, 2020). Pandemic dreamers also describe more bizarre, realistic and vivid dream content (Gorgoni et al., 2021; Iorio et al., 2020). These changes in dreams reflect the increased presence of waking‐day pandemic stressors, such as social isolation, death‐related news and economic repercussions (Iorio et al., 2020; Schredl & Bulkeley, 2020). However, most pandemic dream research has utilized cross‐sectional designs with dream changes quantified using Likert scale items. More detailed content or thematic analysis of recent dream reports has been used in some studies (Barrett, 2020; Giovanardi et al., 2021; Gorgoni et al., 2021); however, they have not asked participants to describe, in their own words, changes to their dream experiences. Therefore, more rich, in‐depth qualitative analysis is required to comprehensively define changes in dreaming during the pandemic.

The COVID‐19 pandemic has also impacted other aspects of sleep health (Kocevska, Blanken, Van Someren, & Rosler, 2020; Varma, Burge, Meaklim, Junge, & Jackson, 2021; Yuksel et al., 2021). Rates of insomnia symptoms increased during the pandemic (Mandelkorn et al., 2020; McCall, Mensah‐Bonsu, Withers, & Gibson, 2021; Morin et al., 2021), with both an increase in new‐onset insomnia symptoms and a worsening of pre‐existing insomnia symptoms observed (Drager et al., 2022; Kocevska et al., 2020; McCall et al., 2021; McCarthy, DeViva, Na, & Pietrzak, 2021; Meaklim, Junge, Varma, Finck, & Jackson, 2021). During the early stages of the pandemic, individuals with insomnia reported higher dream recall frequency, bizarreness, negative emotional tone and more frightening nightmare content compared with good sleepers (Fränkl et al., 2021; Gorgoni et al., 2021; Kennedy et al., 2021). Pre‐pandemic literature suggests that individuals with insomnia experience more nightmares, with more negatively toned, bizarre and health‐related dreams than good sleepers (Feige et al., 2018; Ohayon, Morselli, & Guilleminault, 1997; Sandman et al., 2015, & Schredl, 2009; Schredl, Schäfer, Weber, & Heuser, 2015). Individuals with insomnia experience more dreams related to waking life stressors than good sleepers, likely reflecting their increased perception of, and rumination about, daily stressors (Fernández‐Mendoza et al., 2010; Schredl et al., 1998). Indeed, individuals with insomnia, particularly new‐onset symptoms, experienced higher perceived stress, anxiety and depressive symptoms than good sleepers and those with pre‐existing insomnia in the early stages of the pandemic (Meaklim et al., 2021). As more dream changes have been related to greater personal distress during the COVID‐19 pandemic (Schredl & Bulkeley, 2020), individuals with new‐onset insomnia symptoms may be more vulnerable to negative pandemic dream experiences, compounding their daytime stress and mental health symptoms. As far as the authors are aware, no studies have investigated pandemic dream changes between individuals with new‐onset insomnia symptoms, pre‐existing insomnia symptoms or good sleepers, nor whether these dream changes persist and have implications for sleep and mental health over time.

The current study used a mixed‐methods design to explore: (1) how dream experiences changed during the early stages of the COVID‐19 pandemic using qualitative methods; (2) whether individuals with insomnia symptoms, particularly new‐onset insomnia symptoms compared with pre‐existing insomnia symptoms, experienced more negative dream changes than good sleepers during the COVID‐19 pandemic; (3) if early pandemic dream changes persisted over time; and (4) whether individuals who experienced dream changes throughout the pandemic experienced worse insomnia and mental health symptoms over time.

## 2 METHODS

### 2.1 Participants and procedures

A global online survey captured participants' experiences of insomnia and dreaming during the COVID‐19 pandemic. Individuals aged ≥ 18 years were invited to participate in the survey, hosted by Qualtrics and shared via social media. The baseline survey captured data during the early stages of the COVID‐19 pandemic between 6 April and 15 May 2020 (Meaklim et al., 2021). Follow‐up surveys were conducted at 3‐, 6‐ and 12‐month intervals, concluding in June 2021. Participants were categorized at baseline according to their self‐reported insomnia symptoms to the following question: “Have you ever had or are you currently experiencing insomnia symptoms? (e.g., difficulty falling asleep, staying asleep or waking up too early).” If participants endorsed insomnia symptoms, they were asked whether their insomnia symptoms started before or during the COVID‐19 pandemic. Participant groups were labelled as follows: (1) new‐onset insomnia symptoms (i.e. insomnia symptoms commencing during the pandemic); (2) pre‐existing insomnia symptoms (i.e. insomnia symptoms commencing before the pandemic); (3) good sleepers (i.e. individuals without a history of insomnia symptoms). Although we did not use the Sleep Condition Indicator (SCI) with group participants, SCI total scores and duration of symptoms reported on Item 8 supported participant categorization (Table 1). The institutional Human Research Ethics Committee approved the study protocol.

**TABLE 1:** Participant baseline demographics (*N* = 2240)

	Means ± *SD* or frequencies (%)			
Demographics	Overall group (*N* = 2240)	New‐onset insomnia (*N* = 758)	Pre‐existing insomnia (*N* = 597)	Good sleepers (*N* = 885)
Age (*n* = 2001)	45.51 (± 13.7)	44.1 ± 12.9	44.9 ± 14.8	47.1 ± 13.5
Sex: Female (*n* = 2240)	1613 (72.5)	552 (72.3)	406 (68.0)	655 (74.0)
Marital status: married (*n* = 2229)	1118 (50.2)	368 (48.8)	272 (45.8)	478 (54.3)
Country of residence (*n* = 2240)				
UK	812 (36.7)	141 (18.6)	150 (25.1)	530 (59.9)
South Africa	377 (16.8)	191 (25.2)	152 (18.9)	45 (5.1)
Australia	242 (10.8)	85 (11.2)	100 (16.8)	58 (6.4)
India	268 (12.0)	88 (11.6)	59 (9.9)	121 (13.7)
Ireland	103 (4.6)	47 (6.2)	27 (4.5)	29 (3.2)
Other	429 (19.2)	206 (27.2)	120 (20.1)	103 (11.6)
Currently in lockdown (*n* = 2240)	2096 (93.6)	719 (94.9)	551 (92.3)	826 (93.3)
Employed full‐time (*n* = 2230)	1055 (47.1)	380 (50.1)	246 (41.2)	429 (48.6)
College educated (*n* = 1977)	1559 (78.9)	527 (79.1)	384 (72.7)	648 (82.8)
History of physical health condition (*n* = 2240)	251 (11.2)	90 (11.9)	80 (13.4)	81 (9.2)
History of mental health condition (*n* = 1796)	494 (24.9)	172 (25.8)	164 (31.8)	153 (19.3)
Depression	136 (14.1)	111 (14.6)	108 (18.1)	97 (11.0)
Anxiety	200 (8.9)	60 (4.9)	79 (13.2)	61 (6.9)
Post‐traumatic stress disorder	40 (1.8)	14 (1.8)	17 (2.8)	9 (1.0)
Bipolar disorder	27 (1.2)	16 (2.1)	9 (1.5)	2 (0.2)
Other	77 (3.4)	31 (4.1)	22 (3.7)	24 (2.7)
Currently taking sleep medications (*n* = 2240)	202 (9.0)	88 (11.6)	95 (15.9)	19 (2.1)
Experienced changes in dreams (*n* = 2240)	1006 (44.9)	418 (55.1)	266 (44.6)	322 (36.4)
Insomnia (SCI)^a^	19.30 ± 8.89	13.95 ± 6.11	14.00 ± 6.94	27.44 ± 5.08
Stress (PSS‐10)	18.41 ± 7.60	21.60 ± 6.50	19.85 ± 7.54	14.69 ± 6.91
Anxiety (STAI‐6)	40.41 ± 14.81	46.56 ± 13.98	43.25 ± 14.77	33.30 ± 12.37
Depression (PHQ‐9)	8.30 ± 6.41	11.28 ± 6.10	10.13 ± 6.26	4.52 ± 4.64

*Note*: Countries with the top five number of respondents are listed here, with participants from all other countries listed as “other”.

Abbreviations: PHQ‐9, Patient Health Questionnaire‐9 item; PSS‐10, Perceived Stress Scale‐10 item; SCI, Sleep Condition Indicator (with lower scores representing worse insomnia symptoms and higher scores reflecting better sleep); STAI‐6, State–Trait Anxiety Inventory‐6 Item.

^a^ Median insomnia symptom durations reported on Item 8 of the SCI across groups were: (1) new‐onset insomnia = 1–2 months; (2) pre‐existing insomnia = > 1 year; and (3) good sleepers = I do not have a problem/< 1 month.

### 2.2 Survey

The survey captured information about participant demographics; pandemic‐related lifestyle changes; sleep and insomnia symptoms; and a question regarding changes in dream and nightmare experiences during the COVID‐19 pandemic with a free text option (“Have you experienced any changes in your dreams or nightmares since the COVID‐19 crisis began? (Yes/No); If yes, please provide some more detail if you feel comfortable”). Validated questionnaires were used to assess insomnia (SCI; Espie et al., 2014), stress (Perceived Stress Scale [PSS]; Cohen, Kamarck, & Mermelstein, 1994), anxiety (State–Trait Anxiety Inventory‐6 Item [STAI‐6]; Marteau & Bekker, 1992) and depression (Patient Health Questionnaire‐9 [PHQ‐9]; Kroenke, Spitzer, & Williams, 2001).

### 2.3 Data analysis

Descriptive data characterized the sample and outlined differences between groups using SPSS v26 (IBM) and R (R Core Team, 2020). Qualitative data were processed from open‐ended responses to dreams and nightmare experiences during the pandemic.

#### 2.3.1 Aim 1

Reflexive thematic analysis was used to code qualitative data and derive themes regarding changes in dreaming according to published guidelines (Braun & Clarke, 2006, 2019). Participants' open text responses were first read by Author 1 and Author 4 to generate ideas for initial codes. Preliminary thematic coding was conducted by Author 4, yielding 97 initial codes, verified by Author 1. Initial codes were then translated into overarching themes and subthemes, and reviewed by an independent researcher to verify codes, condense themes and resolve disagreements. A thematic map aided the analysis and grouping of codes into independent themes.

#### 2.3.2 Aim 2

Two methods were used to determine whether individuals with new‐onset and pre‐existing insomnia experienced more negative dream changes than good sleepers. First, Linguistic Inquiry Word Count (LIWC) was used to quantify broader common themes from participants’ open‐ended responses about their dream changes using the LIWC 2015 software (Pennebaker et al., 2015). The LIWC dictionary counts words based on whether they belong to pre‐existing psychologically validated categories of words (e.g. sad words could include “grief”, “cry” or “sad”; Pennebaker et al., 2015). The frequency of a word category is measured by the percentage of occurrence of that word category within a string of text, thereby gathering quantitative summary data from qualitative writing. Non‐parametric Kruskal–Wallis Chi‐Squared tests assessed mean rank differences in words categories between groups. To parallel the qualitative thematic analysis, the LIWC word categories of interest were the 30 sub‐categories of psychological processes, including affective processes, biological processes, drives, perceptual processes, personal concerns and social processes (Pennebaker et al., 2015). A Benjamini–Hochberg correction was applied to control for alpha inflation (Benjamini et al., 1995). Dunn's test was used for post hoc testing, with corrections applied within each word category. Second, we descriptively compared the reflexive thematic analysis between groups, comparing the number of codes, subthemes and themes. Researchers were blind to the participant group for the initial coding of data into themes and subthemes.

#### 2.3.3 Aim 3

A chi‐squared test compared the proportion of participants in each group reporting dream changes at baseline. A Cochran's Q test then assessed the proportion of participants (both for the whole sample and within groups) experiencing changes in dreaming across the pandemic, with McNemar's Test used to assess post hoc comparisons.

#### 2.3.4 Aim 4

Mixed ANOVA was used to investigate the relationship of changes in dreaming (yes/no) with insomnia and mental health (depression, anxiety and stress) over time (baseline, 3, 6 and 12 months), and whether these effects differed across insomnia groups (new‐onset, pre‐existing and good sleepers). Interaction multilevel models were fitted. Models included an interaction term between dream response (within‐person variable) and insomnia groups (between‐person variable) as predictor, with insomnia and mental health symptoms as outcomes. All models had a random intercept by participant to address non‐independence and covariates. Covariates included age, sex, employment, finances impacted during COVID‐19, and prior diagnosis of a mental health condition. Post hoc pairwise comparisons were also calculated for effect of dream changes on outcomes for each insomnia group, using Bonferroni correction. R packages “lme4” (model estimation), “lmerTest” (significance testing) and “emmeans” (post hoc analyses) were used with significance set at *α* = 0.05.

## 3 RESULTS

### 3.1 Descriptive results

A total of 2240 participants who responded to the baseline survey item about changes in dreams were included in the analysis (Table 1). Of these, 758 participants reported experiencing new‐onset insomnia symptoms during the pandemic, 597 reported a history of pre‐existing insomnia symptoms and 885 reported no history of insomnia symptoms.

### 3.2 Aim 1: How did dream experiences change during the **COVID** ‐19 pandemic?

**FIGURE 1:**
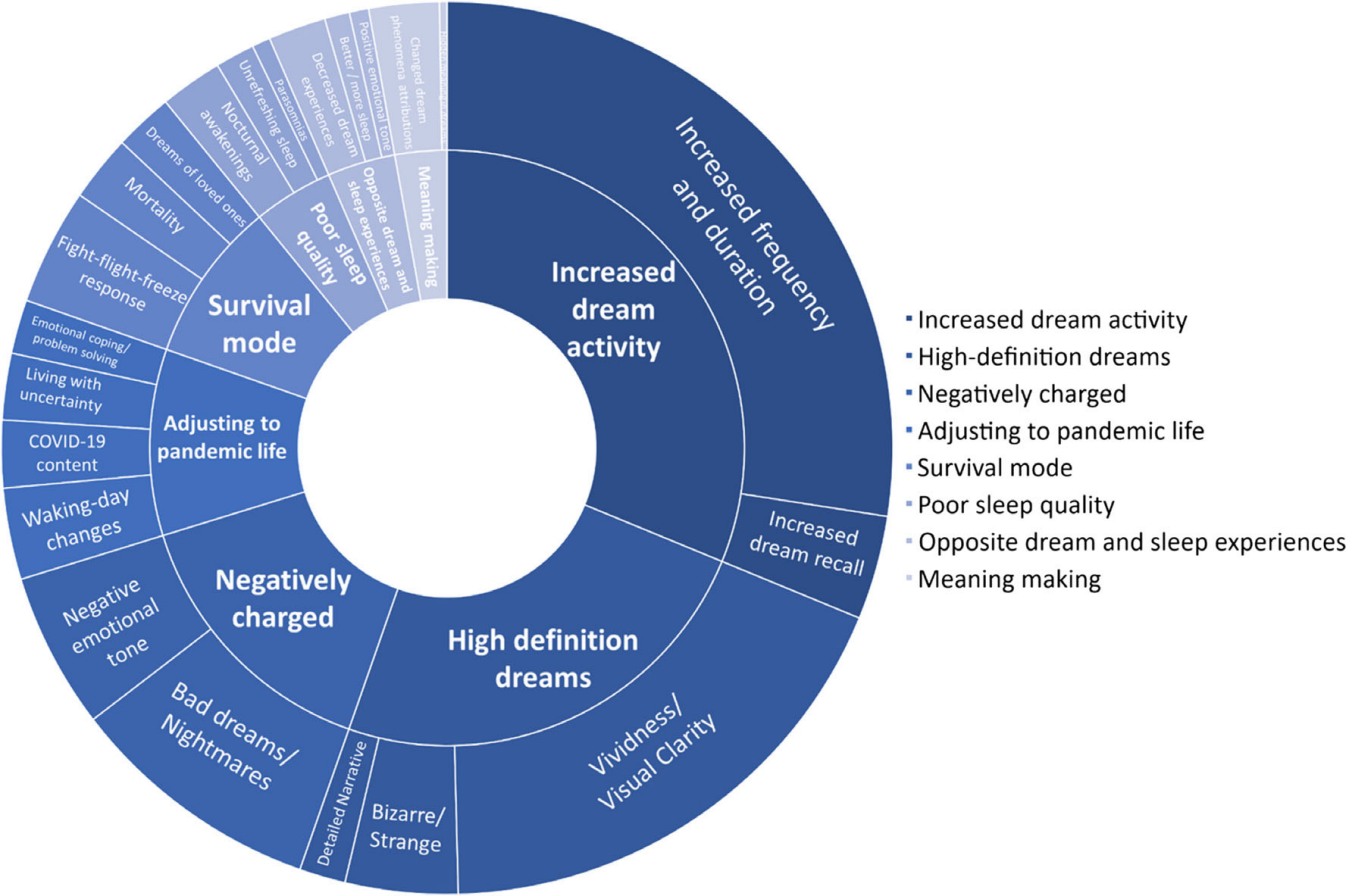
Sunburst diagram outlining the number of codes within pandemic dream change themes/subthemes

Out of 2240 participants, 1006 participants endorsed experiencing dream changes during the pandemic and 911 provided a description of their dream changes for qualitative analysis. Eight themes reflective of changes in dreams and nightmares during the early stages of the pandemic were identified (Figure 1; Table 2).

**TABLE 2:** Themes and subthemes relating to changes in dreams during the COVID‐19 pandemic

Theme	Subtheme
Increased dream activity	Increased frequency and duration
	Increased dream recall
High‐definition dreams	Vividness/visual clarity
	Detailed narrative
	Bizarre/strange
Negatively charged	Bad dreams and nightmares
	Negative emotional tone
Survival mode	Flight‐fight‐freeze response
	Mortality
	Dreams of loved ones
Adjusting to pandemic life	COVID‐19 content
	Waking‐day changes
	Living with uncertainty
	Emotional coping/problem solving
Poor sleep quality	Nocturnal awakenings
	Unrefreshing sleep
	Parasomnias
Meaning‐making	Attributions/explanations for changed dream phenomena
	Hidden meaning
Opposite sleep and dream experiences	Decreased dream experiences
	Better/more sleep
	Positive emotional tone

#### 3.2.1 Theme 1: Increased dream activity

##### Increased frequency and duration

Most participants endorsed experiencing more dreams and nightmares during the pandemic. Increased dream activity was most prevalent early in the pandemic, reducing as the pandemic progressed. Some participants also noted how their dreams had changed in comparison to previous dream experiences (e.g. “[I] prev[iously] didn't dream, now [I have] unpleasant dreams”). Pandemic dream duration also increased, with “dreams that seemed to go on for a long time!!”.

“Just more of it – dreaming – that I can remember. I wake before my alarm at the moment, so I have time to reflect on any dreams or nightmares that I have had.”

##### Increased dream recall

Dreams appeared to be more “memorable”, with participants noting better recall of their dream experiences than usual. Some participants acknowledged that increased dream recall might be due to a recall bias.

“More dream time (or at least remembering them more) and nightmares much more often.”

#### 3.2.2 Theme 2: High‐definition dreams

Pandemic dreams were described in high‐definition with increased vividness and visual clarity. Participants also reported more elaborate dream narratives, with more bizarre or strange qualities.

##### Vividness/visual clarity

Participants reported experiencing more “vivid dreams” during the pandemic with increased visual clarity. For example, participants described dreams that were more “realistic”, “colourful”, “clear” and even “more intense” than usual. Some participants reported experiencing “more lucid dreams”.

“Very vivid dreams that make no sense. But feels so real.”

##### Detailed narrative

Many pandemic dreams had a detailed narrative, with more story‐ or “movie‐like” qualities. Dreams were often “dramatic in nature”, containing more detailed or structured dream content than usual.

“They have more stories, are more complex.”

##### Bizarre/strange

Participants frequently endorsed having more bizarre or strange dreams. The use of language varied considerably, with participants describing their dreams as “bizarre”, “surreal”, “strange”, “odd”, “crazy”, “unusual” and “confusing”.

“Now I remember most of my dreams, they are very strange. …dreams with many details, for example somebody smoking next to me, and I can smell the tobacco burning… (I don't smoke) or cooking and I can smell and taste the flavors….”

#### 3.2.3 Theme 3: Negatively charged

There was an overall negative charge to pandemic dreams, with frequent reports of “bad dreams” and nightmares.

##### Negative emotional tone

Overall, dreams had a negative emotional tone. Some participants also described experiencing negative affect upon awakening after bad dreams, feeling anxious, sad or confused, which impacted their mood during waking hours.

“Unpleasant things that I'm reminded of during the day come more often in my dreams. Bad dreams now affect my mood the entire day.”

##### Bad dreams and nightmares

References to “bad dreams” and “nightmares” were common. Pandemic dreams often contained threatening, dangerous or violent content, with death and loss featuring prominently. Participants also referred to having more “unpleasant dreams”, such as being lonely or seeing monsters or demons.

“Though I have always had bad dreams, those dreams have gotten worse. Maybe because of the ghost rumours around.”

#### 3.2.4 Theme 4: Survival mode

Themes of being in survival mode featured strongly. Dreams made frequent references to the “flight‐fight‐freeze” survival response and highlighted participants' concerns regarding mortality and wishes to connect with loved ones.

##### Flight‐fight‐freeze response

Participants described “frightening dreams”, “anxiety dreams” and “stressful dream scenarios”. Dreams were reported as threatening or dangerous, with violent dream content, often simulating wars or disasters. Participants reported physiological symptoms of the flight‐fight‐freeze response, such as running or being chased, feeling “panicked” with their “heart racing” upon awakening from a bad dream.

“I seem to dream often of falling, being suffocated, or of being helpless to support others in danger – at times, it makes me awake feeling terrified and takes a long time for my heart rate to calm.”

##### Mortality

Themes of death and loss permeated participants' dream reports. Participants reported dreaming about deceased people, either loved ones they had lost or images of dead people in general. Health and illness themes also featured, with dreams about loved ones or themselves becoming sick and dying.

“Woke up crying dreaming of deaths.”

##### Dreams of loved ones

When faced with our mortality, we often remember the ones we love. Participants reported frequently dreaming about family members/relatives, romantic partners and friends. Some dreamt about worries concerning family members' survival, or people they wished to see or had not seen in a long time.

“Family who live far away in UK feature strongly.”

#### 3.2.5 Theme 5: Adjusting to pandemic life

Pandemic dreams reflected how participants' lives had adapted to the pandemic.

##### COVID‐19 content

Dreams specifically containing COVID‐19 content were observed, with dreams detailing COVID‐19 infections, social distancing, lockdown/quarantine, personal protective equipment and death/loss.

“I see Covid‐19 related dreams (l was tested positive and my son kissed me than [sic] l get panicked).”

##### Waking‐day changes

Participants' dream reports were consistent with changes in their waking‐day lives. For example, dreams were related to events that occurred in participants' day‐to‐day lives, such as work, finances, health and emotional stressors.

“My dreams are based on what's happening currently. In our country, South Africa, alcohol is banned. In my dreams, I dream of going to the shops to get wine and then realizing it's banned. Even in my dreams, I am on [sic] lockdown.”

##### Living with uncertainty

Pandemic dreams reflected a state of living with uncertainty. For example, participants reported dreams in which they felt anxious, unsafe and uncertain, without a sense of control.

“Nightmares and recurrent dreams of uncertainty.”

##### Emotional coping/problem‐solving

Participants reported dreams that reflected emotional coping and problem‐solving skills. For example, despite the social isolation caused by lockdowns, participants reported more “relationship‐related” dreams. Dreams also presented a way to “revisit and solve issues”, such as gaining control, stability and searching. Dreams provided an opportunity to emotionally escape from the pandemic, with participants reporting dreams about past memories and future events.

“I've been more lonely and dream about an imaginary romantic partner often.”

#### 3.2.6 Theme 6: Poor sleep quality

Participants frequently referenced poor sleep quality, such as nocturnal awakenings, unrefreshing sleep and parasomnias.

##### Nocturnal awakenings

Participants often reported awakening after experiencing vivid dreams. In some cases, participants advised that vivid or disturbing dreams caused their wakefulness.

“They're more vivid than usual. Now I usually wake up after experiencing a vivid dream.”

##### Unrefreshing sleep

Some participants reported experiencing non‐restorative sleep as a consequence of dreaming. Some also reported that they experienced negative affect (e.g. feeling anxious) or stress upon awakening, instead of being well‐rested.

“Vivid dreams nightly, I wake feeling very unrested.”

##### Parasomnias

Participants reported increases in sleep‐talking, sleep‐walking and “night terrors”. Others noted sleep paralysis and hypnogogic/hypnopompic hallucinations.

“More dreams, very vivid, unusual sense of being awake while dreaming, e.g. conscious but not able to react/respond/wake up.”

#### 3.2.7 Theme 7: Meaning‐making

A theme of meaning‐making was noted, with participants trying to make sense of their changed dream experiences.

##### Attributions/explanations for changed dream phenomena

Participants outlined their thoughts about changes to their dream phenomena. For example, one participant cited increased stress as the culprit, stating that “I always notice a spike in nightmares at time[s] of stress”. Increased anxiety and exposure to social media were also reported as triggers for changed dreaming. Other participants attributed more vivid dreams to increased rapid eye movement (REM) sleep (e.g. “woke up mid REM”). Some participants made reference to sleep extension and experiencing more “morning dreams”.

“Had several nightmares to begin with, weird stuff like family displacement. After getting through the virus and my limiting exposure to social media and the news it stopped.”

##### Hidden meaning

Several participants took a more interpretive approach to understand their changed dream experiences. Pandemic dreams were interpreted as “prophetic” or reflecting a struggle in the person's waking life. One noted that pandemic dreams seemed “easier to interpret”.

“Far more vivid dreams. Sometimes very utopian, as if compensating for my dystopian waking life.”

#### 3.2.8 Theme 8: Opposite dream and sleep experiences

Whilst most participants reported increased dream activity, more negative dreams and poorer sleep quality, this experience was not universal. Some participants alternatively reported decreased dream experiences, better or more sleep, and a positive emotional tone to their dreams.

##### Decreased dream experiences

Some participants reported dreaming “less frequently” and having lower dream recall than usual.

“More nights of dreamless sleep, no nightmares, even the recurring ones haven't been for a while. I can never remember if I dreamed. I used to.”

##### Better/more sleep

Some participants endorsed getting more or better quality sleep.

“At [the] beginning of crisis, I was sleeping more.”

##### Positive emotional tone

Lastly, not all dreams were negatively toned. Some participants endorsed a more positive or neutral emotional tone to their dreams.

“Dreams seem happier, oddly.”

### 3.2 Aim 2: Did individuals with insomnia symptoms experience more negative dream changes during the COVID‐19 pandemic?

Five out of the 30 LIWC word‐categories demonstrated significant group differences (Table 3). Both the new‐onset and pre‐existing insomnia groups used significantly more negative emotion (e.g. hate, worthless) and affect‐related words (e.g. ugly, bitter) to describe changes in dream experiences, compared with good sleepers. In contrast, both insomnia groups used significantly fewer leisure‐related words (e.g. TV, music) than good sleepers. Additionally, individuals with new‐onset insomnia used significantly more anxiety (e.g. nervous, afraid) and death‐related words (e.g. coffin, kill) to describe their changed dream experiences than did good sleepers. No differences in the use of anxiety and death‐related words were noted between insomnia groups (complete LIWC analysis is displayed in Table S1).

**TABLE 3:** LIWC word category differences describing changes in dream experiences between individuals with new‐onset insomnia, pre‐existing insomnia and good sleepers during the COVID‐19 pandemic

			New‐onset insomnia dreams (*n* = 369)	Pre‐existing insomnia dreams (*n* = 240)	Good sleeper dreams (*n* = 302)	New‐onset and pre‐existing insomnia	New‐onset insomnia and good sleepers	Pre‐existing insomnia and good sleepers
Word type	Kruskal–Wallis chi squared test	Adj. *p*‐value	M_rank_	M_rank_	M_rank_	Post hoc Dunn test (*Z*‐value, adj. *p*‐value)	Post hoc Dunn test (*Z*‐value, adj. *p*‐value)	Post hoc Dunn test (*Z*‐value, adj. *p*‐value)
Negative emotion	34.03	< 0.001***	13.80	11.14	6.33	−1.72, *p* = 0.086	−5.78, *p* < 0.001***	−3.54, *p* < 0.001***
Affect words	23.20	< 0.001***	14.34	12.22	7.58	−1.07, *p* = 0.284	−4.71, *p* < 0.001***	−3.20, *p* = 0.002**
Leisure	20.01	< 0.001***	12.94	11.26	16.02	−1.17, *p* = 0.241	3.42, *p* < 0.001***	4.20, *p* < 0.001***
Anxiety	12.37	0.021*	3.87	2.61	1.21	−1.52, *p* = 0.128	−3.52, *p* = 0.001**	−1.70, *p* = 0.128
Death	10.33	0.031*	1.02	0.60	0.46	−1.56, *p* = 0.169	−3.20, *p* = 0.004**	−1.38, *p* = 0.169

*Note*: This table displays mean rank differences of the significant LIWC word category differences describing changes in dreaming between groups.

*** *p* < 0.001.

** *p* < 0.01.

* *p* < 0.05, adj. *p*‐value = adjusted *p‐*value using the Benjamini and Hochberg (1995) correction for multiple comparisons, where critical value cut‐off scores were converted to adjusted *p*‐values using the method described by Benjamini, Heller, Yekutieli (2009). For Dunn's test post hoc comparisons, corrections were applied within each word category, not all word categories. Only results that reached statistical significance are displayed in this table. Please refer to Table S1 for 30 LIWC category results.

To put the LIWC analysis in the context of the thematic analysis, we descriptively explored differences in dream themes/subthemes between groups. Consistent with the LIWC analysis, the new‐onset (58%) and pre‐existing insomnia (52%) groups reported more negatively charged dream themes than did the good sleeper groups (30%). In particular, a greater proportion of bad dreams and nightmares were endorsed by the new‐onset (36%) and pre‐existing insomnia (33%) groups than good sleepers (19%). Of note, the new‐onset insomnia group reported more themes related to survival mode (35%) and poor sleep quality (38%) than the other groups (Table S2).

### 3.3 Aim 3: Did changed dream experiences continue throughout the COVID‐19 pandemic?

A significant association between insomnia group and changed dream experiences was observed at baseline (*χ* ^2^ [2, *n* = 2240] = 58.13, *p* < 0.001, Cramer's V = 0.161). A higher proportion of individuals with new‐onset insomnia reported experiencing dream changes during the pandemic (55%) compared with those with pre‐existing insomnia (45%; *p* < 0.001) and good sleepers (36%; *p* < 0.001). Individuals with pre‐existing insomnia also reported a significantly higher proportion of changed dream experiences than good sleepers (*p* = 0.002).

**FIGURE 2:**
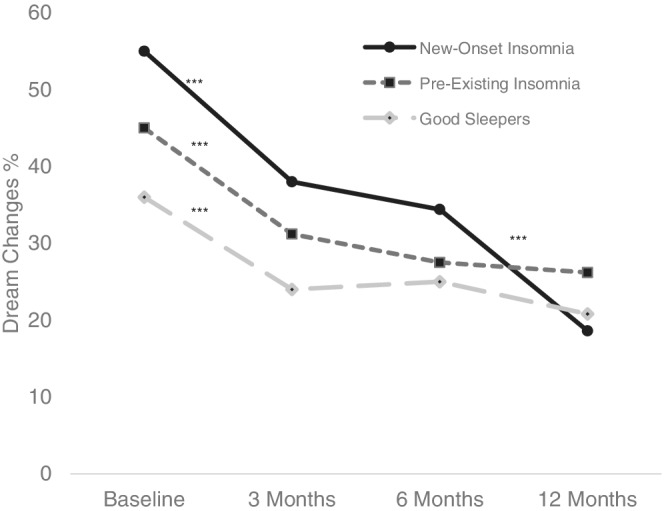
The proportion of participants experiencing changes in dreaming across insomnia groups during the COVID‐19 pandemic. ***p < 0.001; the baseline survey enquired about changes in dreams/nightmares since the pandemic started, whereas the follow‐up surveys inquired about increases in dreams/nightmares over the past month, compared with pre‐pandemic levels. Total sample size decreased from baseline (*n* = 2240) to 3 months (*n* = 970), but remained stable from 6 (*n* = 761) to 12 months (*n* = 766)

There was a significant reduction in the proportion of participants endorsing dream changes over 12 months (*χ* ^2^ [3, *n* = 468] = 58.13, *p* < 0.001; Figure 2). Post hoc tests indicated a significant decrease in dream activity from baseline to 3 months (*p* < 0.001) and from 6 to 12 months (*p* = 0.003), with no change from 3 to 6 months (*p* = 0.717). Within groups, the new‐onset insomnia group had a significant reduction in dream activity from baseline to 3 months (*p* < 0.001) and 6 to 12 months (*p* < 0.001), but not from 3 to 6 months (*p* = 0.336). The reduction in dream activity from 6 to 12 months did not coincide with an improvement in insomnia symptoms during this time (Figure S1). Dream activity for the pre‐existing insomnia and good sleeper groups reduced significantly from baseline to 3 months only (*p* < 0.001).

We performed a sensitivity analysis to compare dream/nightmare changes between males and females. We did note a higher proportion of females (48%) reporting changes in their dreams/nightmares at baseline than males (36%). However, this sex difference reduced over time (Table S3). Additionally, when we examined differences in the proportion of males/females experiencing dream/nightmare changes across our insomnia groups, we found a similar pattern for both males and females. Individuals experiencing new‐onset insomnia at baseline, regardless of sex, had the highest proportion of dream/nightmare changes at baseline than those with pre‐existing insomnia or good sleepers (all *p* < 0.001), with insomnia group differences between sexes reducing over time (Table S4).

### 3.4 Aim 4: Did changed dream experiences have any impact on insomnia and mental health symptoms throughout the pandemic?

Overall, there were significant main effects of changes in dream experiences on all insomnia and mental health outcomes over time, such that individuals who reported dream changes had worse symptoms than those with no changes (*p* < 0.001). There were also significant dream changes × insomnia group interaction effects on insomnia and depression. Individuals with changes in dreaming differed in their insomnia and depressive symptoms (*p* < 0.001 and *p* = 0.006, respectively) from those without changes, and this effect was different between insomnia groups. No significant interaction effects emerged for stress or anxiety (*p* = 0.66 and *p* = 0.42, respectively). Effect sizes were small (*ω*_p_^2^ ranging from 0.005 to 0.02) to negligible (*ω*_p_^2^ < 0.01).

**FIGURE 3:**
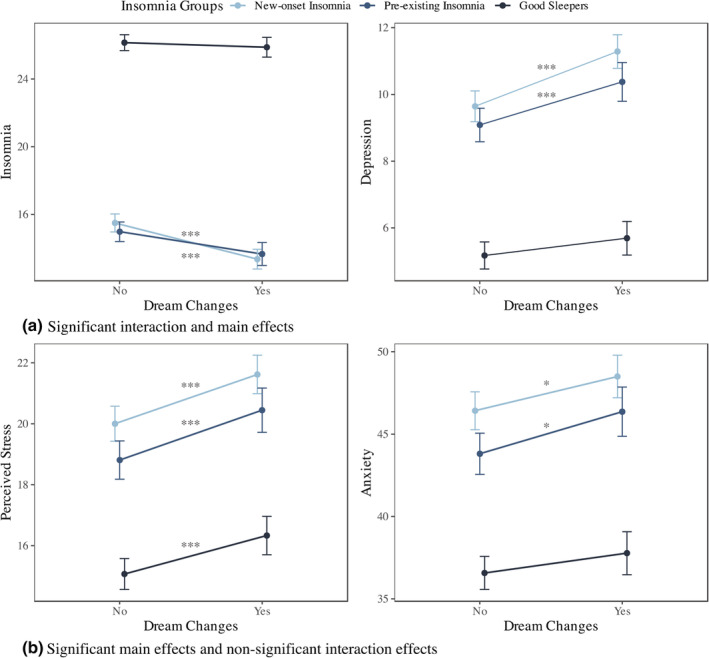
Insomnia and mental health throughout the pandemic between people with and without changes in dreams for each insomnia group. *p < 0.05, ***p < 0.001. Interactions effect and main effects of dreams changes were significant for insomnia and depression (a). Main effects, but not interaction effects, were significant for stress and anxiety (b)

Differences in insomnia and mental health between individuals with and without changes in dreams for each insomnia group are displayed in Table 4 and Figure 3. For individuals with pre‐existing and new‐onset insomnia, those who reported changes in dreaming experienced more insomnia and mental health symptoms than those who reported no changes (all *p* < 0.05). In contrast, good sleepers with changes in dreaming differed from those without changes in stress (*p* < 0.001), but not anxiety or depression (all *p* > 0.05).

**TABLE 4:** Effects of changes in dreaming on insomnia and mental health over time, and estimated mean difference between individuals with and without change in dreams across insomnia groups

Symptom	Pairwise comparison				
	Insomnia group				
	New‐onset insomnia	Pre‐existing insomnia	Good sleepers	Interaction effects (dream × insomnia group)	Main effects (dream changes)
Insomnia	**2.14** *** **[1.29, 2.99]**	**1.31** *** **[0.35, 2.28]**	0.27 [−0.56, 1.10]	***F***_**2,3528**_ **= 10.15** *** **, 0.005**	***F***_**1,3510**_ **= 48.83** *** **, 0.010**
Stress	**−1.62** *** **[−2.54, −0.69]**	**−1.64** *** **[−2.67, −0.60]**	**−1.26** *** **[−2.16, −0.37]**	*F*_2,3527_ = 0.42, 0.00	***F***_**1,3509**_ **= 60.56** *** **, 0.020**
Depression	**−1.64** *** **[−2.38, −0.91]**	**−1.29** *** **[−2.12, −0.46]**	−0.52 [−1.23, 0.20]	***F***_**2,3461**_ **= 5.15** ** **, 0.002**	***F***_**1,3444**_ **= 56.10** *** **, 0.020**
Anxiety	**−2.08** * **[−4.12, −0.04]**	**−2.56** * **[−4.86, −0.26]**	−1.20 [−3.19, 0.78]	*F*_2,3787_ = 0.87, 0.00	***F***_**1,3773**_ **= 20.66** *** **, 0.005**

*Notes*: Values presented for pairwise comparison are estimated differences and [95% confidence intervals]. Positive values indicate that people who reported changes in dreams had lower scores, compared with those who reported no dream changes. Conversely, negative values show that people with changes in dreams had higher scores than people who reported no dream changes. Values presented for interaction and main effects are *F*‐statistics, degree of freedom and *ω*_p_^2^ effect sizes. Boldface highlights adjusted *p* < 0.05. The following covariates were included in adjusted models: age, sex, employment, finances impacted during COVID‐19, and prior diagnosis of a mental health condition.

* *p <* 0.05.

** *p <* 0.01.

*** *p <* 0.001.

## 4 DISCUSSION

This study used a mixed‐methods design to examine dream changes between individuals with and without insomnia symptoms and their relationship to mental health during the COVID‐19 pandemic. Overall, participants endorsed increased dream activity during the early stages of the pandemic, often dreaming vividly, in high‐definition and with a strong negative charge. Themes related to survival, meaning‐making and adjusting to pandemic life were also noted. A higher proportion of individuals with insomnia symptoms experienced changes in their dreams than good sleepers, using more negative and affect‐related words, with fewer leisure‐related words, to describe their dream changes. Specifically, individuals with new‐onset insomnia that developed during the pandemic reported more distressing dream changes than those who slept well, using more anxious and death‐related words than those who slept well. All groups reported a significant decline in dream activity from 3 months to each follow‐up point, with the new‐onset insomnia group experiencing another reduction in dream activity from 6 to 12 months. Individuals with insomnia symptoms who experienced dream changes reported worse mental health symptoms throughout the pandemic than those who did not experience dream changes. Together, our unique longitudinal mixed‐methods approach emphasizes that individuals with insomnia symptoms, particularly new‐onset insomnia, were more prone to changes in dreams and negative dreams experiences during the COVID‐19 pandemic than those who slept well. Dream changes and insomnia symptoms may therefore be important markers of distress and mental health during collective stressful events, such as a global pandemic.

Our qualitative data demonstrate that dream experiences changed vastly during the early stages of the pandemic. Overall, dream activity increased during the pandemic, consistent with other dream research (Gorgoni et al., 2021; Pesonen et al., 2020; Scarpelli et al., 2021). Dreams were described in high‐definition, not only more vivid, strange and bizarre (Gorgoni et al., 2021; Iorio et al., 2020), but with increased visual clarity and detailed storylines. Dreams also highlighted how people adjusted to pandemic life; participants dreamt about COVID‐19, changes to their waking‐day lives, and ways to cope and problem solve. In addition, pandemic dreams were negatively charged, with participants endorsing more bad dreams and nightmares, in line with other pandemic research (Barrett, 2020; Scarpelli et al., 2021). Unique to our study, participants reported being in survival mode, with dreams containing references to the flight‐fight‐freeze response, mortality and thoughts of loved ones, outlining the real threat to survival posed by the COVID‐19 pandemic. Poor sleep quality was a common theme throughout participants' dream reports, consistent with Scarpelli et al. (2021), who found that poor sleep predicts increased nightmare frequency. Our quantitative data support this, with more individuals with insomnia experiencing pandemic‐related dream changes and nightmares than good sleepers. These data add qualitative depth to recent pandemic dream research, outlining the extent of dreams changes and their relationship to poor sleep quality and insomnia symptoms during the COVID‐19 pandemic.

Insomnia symptoms were associated with more negative dream experiences during the early stages of the pandemic, consistent with pre‐ and early‐pandemic literature reporting that insomnia is associated with increased stress perception and negative dream emotionality reflecting current stressors (Fernández‐Mendoza et al., 2010; Meaklim et al., 2021; Schredl et al., 1998). A novel finding of our study is that individuals with insomnia symptoms developing during the pandemic were most affected, reporting the highest proportion of dream changes, coupled with more anxious and death‐related dreams, reflecting a state of survival mode. Therefore, individuals who experienced insomnia symptoms during the pandemic, particularly new‐onset insomnia, were more vulnerable to dream‐related distress and negativity than those who slept well.

Insomnia symptoms were related to more dream changes and negative affect, raising important questions about the underlying mechanisms linking dreaming and insomnia. Theoretically, individuals with insomnia symptoms appear to be more affected by stressful events, and are generally more stressed, anxious or threat‐sensitive than good sleepers (Akram, Barclay, & Milkins, 2018; Fernández‐Mendoza et al., 2010; Healey et al., 1981; Perogamvros, Castelnovo, Samson, & Dang‐Vu, 2020; Roth & Drake, 2004). Daytime anxiety is also associated with negative dream affect (Sikka, Pesonen, & Revonsuo, 2018). Given that the new‐onset insomnia group had the highest stress and anxiety levels, they may perceive the pandemic as more threatening and anxiety‐provoking, thereby increasing the frequency and severity of anxiety and death‐related themes in dreams, consistent with both the continuity hypothesis and threat simulation theory of dreaming (Revonsuo, 2000; Schredl et al., 1998; Sikka et al., 2018). Dream changes and insomnia symptoms may therefore be evolutionary mechanisms to keep us awake and safe in times of danger (Perogamvros et al., 2020; Revonsuo, 2000), but may not offer an evolutionary advantage for modern‐day crises, like a global pandemic.

Alternatively, the poor sleep quality experienced by individuals with insomnia symptoms may have increased pandemic dream recall. Our finding that individuals with insomnia symptoms reported more dream changes than good sleepers supports the arousal‐retrieval model of dream recall, which proposes that increased sleep fragmentation and awakenings assist dream recall (Koulack & Goodenough, 1976; Scarpelli et al., 2021). Our findings also support the REM instability hypothesis, which theorizes that increased arousal in insomnia leads to a reduction and fragmentation of REM sleep, the stage of sleep where more bizarre and emotional dreams occur, with the experience of non‐restorative sleep leading to improved dream recall, especially for emotionally charged content (Feige et al., 2018; Riemann et al., 2012; Riemann, Krone, Wulff, & Nissen, 2020; Zadra & Stickgold, 2021). Increased activation of the default mode network (DMN) is associated with REM sleep and dreaming, and alterations to the DMN are observed in individuals with insomnia (Kay et al., 2016; Zadra & Stickgold, 2021), which may account for associations between increased dream activity and insomnia. In contrast, associations between longer sleep duration and increased dream recall have also been reported during the pandemic (Scarpelli et al., 2021). This discrepancy may be explained by the naturalistic sleep extension that occurred during the COVID‐19 pandemic (Bottary, Simonelli, Cunningham, Kensinger, & Mantua, 2020; Yuksel et al., 2021), also noted in our qualitative findings. As people extended their sleep beyond habitual levels and satiated any homeostatic sleep debt, their sleep may have become more fragmented and increased dream recall (Bottary et al., 2020). More work is needed to uncover underlying brain regions and REM sleep mechanisms that link insomnia and dream activity.

Bidirectional relationships likely exist between dreaming, insomnia, negative affect and mental health. As REM sleep contributes to restoring optimal emotional brain reactivity to cope with negative events, insomnia with unstable REM sleep may impair the overnight resolution of distress, leading to increased daytime anxiety and stress (Deliens, Gilson, & Peigneux, 2014; Wassing et al., 2016). This lack of “overnight therapy” from stable REM sleep (Walker & van Der Helm, 2009) may explain why individuals with insomnia symptoms and dream changes in our study reported worse mental health symptoms throughout the pandemic. More research is warranted to understand how dream activity and insomnia symptoms contribute to poor mental health.

These findings should be considered in light of some limitations. We grouped participants based on their responses to two survey items regarding self‐reported insomnia symptoms and symptom onset in relation to the pandemic, and not using a validated insomnia measure. Whilst SCI total scores and symptom duration on Item 8 supported participant groupings, the SCI cut‐off score for insomnia disorder (SCI ≤ 16) is not currently validated to distinguish acute insomnia from insomnia disorder. Also, clinical interviews could not be conducted due to the size and speed of study rollout. As a third of our sample reported a duration of insomnia symptoms of < 3 months, using the SCI cut‐off score for insomnia disorder to determine participant groupings was inappropriate. Therefore, future validation studies of the SCI to determine cut‐off scores inclusive of symptom duration to distinguish acute insomnia from insomnia disorder, and cut‐off scores for insomnia symptoms versus insomnia disorder are recommended.

It is important to note that participants' insomnia symptoms may have changed over time. However, mean SCI scores remained consistent over time (Figure S1). Additionally, we did see a small sex difference in pandemic dream recall early in the pandemic, similar to previous studies (Barrett, 2020; MacKay & DeCicco, 2020; Scarpelli et al., 2021). However, with unequal proportions of males and females in the sample it is difficult to determine whether this is a true result or a sampling issue. Future dream research like this should aim to recruit equal proportions of males and females to investigate this potential sex difference further. In addition, a reduction in sample size was observed from baseline to 3‐month follow‐up, but was minimal from 6 to 12 months. Although we did not use validated past dream methodology such as Likert scales to reflect positive/negatively valenced dreams, asking an open‐ended free response about dream/nightmare changes is a unique strength of our research that adds additional information to the dreaming experiences during the COVID‐19 pandemic to support existing, validated techniques. Lastly, LIWC does not account for negations to word categories (e.g. “happy” versus “not happy”) as it provides frequency or word category types that appear in a body of text (i.e. they are count data). Therefore, it is important to interpret the findings of the LIWC alongside the study's qualitative findings.

In conclusion, dream activity increased in the early stages of the COVID‐19 pandemic but reduced significantly over time. Changed dreams were associated with worse insomnia symptoms and mental health symptoms over time, with individuals with new‐onset insomnia symptoms experiencing more dream changes and negative dream experiences focused on survival themes than good sleepers. These results highlight the need for mental health clinicians to inquire about insomnia and dream changes during times of crisis. These findings will hopefully contribute to more effective mental health recovery from the global pandemic, and ultimately inform novel design of treatments for both insomnia and mental health.

## CONFLICTS OF INTEREST

The authors declare no conflict of interest. Hailey Meaklim is supported by an Australian Government Research Training Program Scholarship administered through Monash University.

## AUTHOR CONTRIBUTIONS

Conception or design of the work: HM, MFJ, PV and MLJ. Data collection: HM, PV, WS and SG. Data analysis and interpretation: HM, SKB, MB, FL, MLJ and ICR. Drafting the article: HM, SKB, MLJ, FL, MB and IR. Critical revision of the article: HM, MB, FL, PV, MLJ, ICR, WS and SG. Final approval of the version to be published: HM and MLJ.

## Supporting information


**TABLE S1** Word category differences describing changes in dream experiences between individuals with new‐onset insomnia, pre‐existing insomnia and good sleepers during the COVID‐19 pandemic
**TABLE S2** Pandemic dream themes and subthemes endorsed across insomnia groups (number of codes making up subtheme/theme with % of codes per insomnia group in brackets)
**FIGURE S1** Sleep Condition Indicator scores across insomnia groups over 12 months of the COVID‐19 pandemic
**TABLE S3** Proportion males/females experiencing dream changes across the pandemic
**TABLE S4** Proportion of participants experiencing dream/nightmare changes by insomnia group and sex

## Data Availability

The data that support the findings of this study are available from the corresponding author upon reasonable request.
