# Supplementary material for: Strange themes in pandemic dreams: Insomnia was associated with more negative, anxious and death‐related dreams during the COVID‐19 pandemic
Source: J Sleep Res. 2022 Jun 14:e13655. Online ahead of print. doi: 10.1111/jsr.13655 (PMC9350044; doi:10.1111/jsr.13655)
Supplement: Supplementary file 1 — TABLE S1 Word category differences describing changes in dream experiences between individuals with new‐onset insomnia, pre‐existing insomnia and good sleepers during the COVID‐19 pandemic TABLE S2 Pandemic dream themes and subthemes endorsed across insomnia groups (number of codes making up subtheme/theme with % of codes per insomnia group in brackets) FIGURE S1 Sleep Condition Indicator scores across insomnia groups over 12 months of the COVID‐19 pandemic TABLE S3 Proportion males/females experiencing dream changes across the pandemic TABLE S4 Proportion of participants experiencing dream/nightmare changes by insomnia group and sex [file JSR-9999-0-s001.docx]

**Online Supplement**

**Table S1**

*Word category differences describing changes in dream experiences between individuals with new-onset insomnia, pre-existing insomnia and good sleepers during the COVID-19 pandemic.*

|  |  |  | **New Onset Insomnia Dreams**  **(*n* = 369)** | **Pre-existing Insomnia Dreams**  **(*n* = 240)** | **Good Sleeper Dreams**  **(*n =* 302)** | **New Onset and Pre-existing Insomnia** | **New Onset Insomnia and Good Sleepers** | **Pre-existing Insomnia and Good Sleepers** |
| --- | --- | --- | --- | --- | --- | --- | --- | --- |
| **Word type** | **Kruskal Wallis Chi Squared Test** | **Adj. *p*-value** | ***M_rank_*** | ***M_rank_*** | ***M_rank_*** | **Post-hoc Dunn Test (*Z*-value,**  **adj. *p*-value)** | **Post-hoc Dunn Test (*Z*-value,**  **adj. *p*-value)** | **Post-hoc Dunn Test**  **(*Z*-value,**  **adj. *p*-value)** |
| Negative emotion | 34.03 | <.001*** | 13.80 | 11.14 | 6.33 | -1.72,  *p* = .086 | -5.78,  *p* < .001*** | -3.54,  *p* < .001*** |
| Affect words | 23.20 | <.001*** | 14.34 | 12.22 | 7.58 | -1.07,  *p* = .284 | -4.71,  *p* < .001*** | -3.20,  *p* = .002** |
| Leisure | 20.01 | <.001*** | 12.94 | 11.26 | 16.02 | -1.17,  *p* = .241 | 3.42,  *p* < .001*** | 4.20,  *p* < .001*** |
| Anxiety | 12.37 | .021* | 3.87 | 2.61 | 1.21 | -1.52,  *p* = .128 | -3.52,  *p* = .001** | -1.70,  *p* = .128 |
| Death | 10.33 | .031* | 1.02 | 0.60 | 0.46 | -1.56,  *p* = .169 | -3.20,  *p* = .004** | -1.38,  *p* = .169 |
| Achievement | 7.63 | .111 | 1.12 | 0.91 | 1.23 |  |  |  |
| Sadness | 6.81 | .141 | 0.82 | 0.26 | 0.21 |  |  |  |
| Positive emotion | 4.42 | .411 | 0.53 | 0.99 | 1.22 |  |  |  |
| Anger | 3.50 | .461 | 0.67 | 0.44 | 0.35 |  |  |  |
| Family | 3.51 | .461 | 0.69 | 0.44 | 0.24 |  |  |  |
| Ingest | 3.53 | .461 | 0.01 | 0.06 | 0.02 |  |  |  |
| Religion | 3.40 | .461 | 0.09 | 0.00 | 0.08 |  |  |  |
| Feel | 2.93 | .531 | 0.48 | 0.37 | 0.13 |  |  |  |
| See | 2.55 | .601 | 8.45 | 8.21 | 9.12 |  |  |  |
| Power | 2.13 | .691 | 1.25 | 0.99 | 0.73 |  |  |  |
| Perception | 1.70 | .801 | 9.09 | 9.16 | 9.53 |  |  |  |
| Health | 1.43 | .831 | 0.73 | 0.59 | 0.35 |  |  |  |
| Home | 1.40 | .831 | 0.36 | 0.37 | 0.13 |  |  |  |
| Social | 0.87 | .831 | 3.67 | 3.26 | 2.82 |  |  |  |
| Friend | 1.03 | .831 | 0.31 | 0.12 | 0.24 |  |  |  |
| Hear | 1.15 | .831 | 0.10 | 0.43 | 0.09 |  |  |  |
| Sexual | 0.81 | .831 | 0.06 | 0.04 | 0.03 |  |  |  |
| Affiliation | 0.91 | .831 | 1.08 | 1.08 | 0.77 |  |  |  |
| Reward | 0.94 | .831 | 0.59 | 0.52 | 0.60 |  |  |  |
| Drives | 0.62 | .841 | 4.86 | 4.10 | 3.62 |  |  |  |
| Work | 0.57 | .841 | 1.30 | 0.77 | 0.80 |  |  |  |
| Money | 0.60 | .841 | 0.09 | 0.01 | 0.07 |  |  |  |
| Risk | 0.46 | .851 | 1.10 | 0.80 | 0.65 |  |  |  |
| Biological process | 0.29 | .901 | 1.52 | 1.60 | 1.38 |  |  |  |
| Body | 0.04 | .981 | 0.66 | 0.80 | 0.87 |  |  |  |
| *Note.* *** = *p* < .001, ** = *p* < .01, * = *p* < .05, adj. *p*-value = adjusted *p-*value using the Benjamini and Hochberg (1995) correction for multiple comparisons, where critical value cut off scores were converted to adjusted *p*-values using the method described by Benjamini, Heller, Yekutieli (2009). For Dunn’s Test post-hoc comparisons, corrections were applied within each word category, not all word categories. | | | | | | | | |

Table S2. Pandemic dream themes and subthemes endorsed across insomnia groups (Number of codes making up subtheme/theme with % of codes per insomnia group in brackets)

| Theme | Subtheme | Overall Group  (*n* = 911) | New Onset Insomnia  (*n* = 369) | Pre-existing Insomnia  (*n* = 240) | Good Sleepers  (*n* = 302) |
| --- | --- | --- | --- | --- | --- |
| Increased dream activity |  | 895 | 326 (88%) | 223 (93%) | 346 (115%) |
|  | Increased frequency/duration | 787 | 293 (79%) | 199 (83%) | 295 (98%) |
|  | Increased dream recall | 108 | 33 (9%) | 24 (10%) | 51 (17%) |
| High-definition dreams |  | 692 | 261 (70%) | 197 (82%) | 226 (75%) |
|  | Vividness/visual clarity | 527 | 201 (54%) | 154 (64%) | 171 (57%) |
|  | Detailed narrative | 48 | 14 (4%) | 13 (5%) | 14 (5%) |
|  | Bizarre/strange | 117 | 46 (12%) | 30 (12%) | 41 (14%) |
| Negatively charged |  | 427 | 214 (58%) | 124 (52%) | 92 (30%) |
|  | Bad dreams and nightmares | 265 | 132 (36%) | 80 (33%) | 56 (19%) |
|  | Negative emotional tone | 113 | 62 (22%) | 29 (18%) | 22 (12%) |
| Adjusting to pandemic life |  | 248 | 101 (27%) | 68 (28%) | 74 (24%) |
|  | COVID-19 content | 28 | 13 (4%) | 8 (3%) | 7 (2%) |
|  | Waking-day changes | 95 | 36 (10%) | 25 (10%) | 34 (11%) |
|  | Living with uncertainty | 69 | 37 (10%) | 18 (8%) | 14 (5%) |
|  | Emotional coping/problem solving | 56 | 15 (4%) | 17 (7%) | 19 (6%) |
| Survival mode |  | 237 | 130 (35%) | 55 (23%) | 52 (17%) |
|  | Flight-fight-freeze response | 111 | 60 (16%) | 27 (11%) | 24 (8%) |
|  | Mortality | 62 | 39 (11%) | 10 (4%) | 13 (4%) |
|  | Dreams of loved ones | 64 | 31 (8%) | 18 (8%) | 15 (5%) |
|  |  |  |  |  |  |
| Poor sleep quality |  | 125 | 141 (38%) | 78 (33%) | 64 (21%) |
|  | Nocturnal awakenings | 65 | 32 (9%) | 14 (6%) | 6% |
|  | Unrefreshing sleep | 41 | 21 (6%) | 10 (4%) | 10 (3%) |
|  | Parasomnias | 19 | 4 (1%) | 7 (3%) | 6 (2%) |
| Opposite sleep/dream experiences |  | 106 | 25 (7%) | 42 (18%) | 43 (14%) |
|  | Decreased dream experiences | 60 | 16 (4%) | 27 (11%) | 17 (6%) |
|  | Better/More Sleep | 26 | 8 (2%) | 7 (3%) | 14 (5%) |
|  | Positive Emotional Tone | 20 | 1 (0%) | 8 (3%) | 12 (4%) |
| Meaning making |  | 80 | 29 (8%) | 20 (8%) | 34 (11%) |
|  | Changed dream phenomena attributions/explanations | 72 | 27 (7%) | 18 (8%) | 30 (10%) |
|  | Hidden meaning | 8 | 2 (0%) | 2 (1%) | 4 (1%) |

Note. More than one code per participant could be coded under a subtheme/theme. Participants’ responses were therefore not mutually exclusive and responses allowed for multiple codes per response; therefore, percentages do not add up to 100%.

**Figure 1.** Sleep Condition Indicator scores across insomnia groups over 12-months of the COVID-19 pandemic

**Difference in proportion of dream changes in males compared to females across the pandemic.**

We did note a higher proportion of dream/nightmare changes in females compared to males at baseline (48.4 vs. 35.8 – Table S3). Also, the discrepancy between males and female dream/nightmare changes decreased over time. There was a significant reduction in the proportion of both male and female participants endorsing dream changes over 12 months (*p*’s <.001)

**Table S3.**

Proportion males/females experiencing dream changes across the pandemic

|  | Baseline | 3 Months | 6 Months | 12 Months |
| --- | --- | --- | --- | --- |
| Male | 597 (35.8%) | 195 (26.7%) | 148 (28.4%) | 147 (23.8%) |
| Female | 1613 (48.4%) | 764 (32.5%) | 604 (29.6%) | 611 (21.1%) |

**Differences in the proportion of dream/nightmares changes in males compared to females across insomnia groups across the pandemic.**

We looked at differences in the proportion of males/females experiencing dream changes across our insomnia categories over time and found a similar pattern for both males and females. Individuals experiencing New-Onset Insomnia at baseline, regardless of sex, had the highest proportion of dream/nightmare changes at baseline than those with pre-existing insomnia or good sleepers (all *p*’s <.001).

There was a significant decrease in the proportion of participants in the new onset insomnia group reporting a change in dreams/nightmares across time (Table S4). Post hoc tests revealed that in the overall group, there was a significant decrease in the proportion of dream/nightmare changes from Baseline to 3 months, and then again from 6 to 12 months. This pattern was noted in female participants, but males in the new onset insomnia group only had a significant decrease in the proportion of dream changes from baseline to 3 months. For the pre-existing insomnia group, there was only a significant decrease in dream changes from baseline to 3 months, and no difference was found for male vs female participants. Lastly, good sleepers also experienced a decrease in the proportion of participants reporting dream changes from baseline to three months. However, there was a sex difference here, with this significant decrease only noted for female participants.

**Table S4.**

Proportion of participants experiencing dream/nightmare changes by insomnia group and sex.

| Insomnia Group | Sex | Baseline  (T0) | 3 Months  (T1) | 6 Months  (T2) | 12 Months  (T3) | Cochranes Q | Post-Hoc |
| --- | --- | --- | --- | --- | --- | --- | --- |
| New-Onset Insomnia | Overall Group | 55 | 38 | 34 | 19 | 46.85*** | T0>T1, T2>T3 |
|  | Males | 49 | 37 | 38 | 24 | 10.08* | T0>T1 |
|  | Females | 58 | 38 | 34 | 17 | 46.26*** | T0>T1, T2>T3 |
| Pre-Existing Insomnia | Overall Group | 45 | 31 | 28 | 26 | 14.91** | T0>T1 |
|  | Males | 37 | 25 | 34 | 30 | 8.58* | T0>T1 |
|  | Females | 49 | 34 | 26 | 26 | 30.72*** | T0>T1 |
| No insomnia symptoms | Overall Group | 36 | 24 | 25 | 21 | 20.36*** | T0>T1 |
|  | Males | 23 | 20 | 15 | 19 | 6.60 | - |
|  | Female | 41 | 25 | 28 | 22 | 34.39*** | T0>T1 |
